# Lentiviral Protein Transduction with Genome-Modifying HIV-1 Integrase-I-PpoI Fusion Proteins: Studies on Specificity and Cytotoxicity

**DOI:** 10.1155/2014/379340

**Published:** 2014-04-22

**Authors:** Vesa Turkki, Diana Schenkwein, Oskari Timonen, Tiia Husso, Hanna P. Lesch, Seppo Ylä-Herttuala

**Affiliations:** ^1^Department of Biotechnology and Molecular Medicine, A.I. Virtanen Institute for Molecular Sciences, University of Eastern Finland, 70210 Kuopio, Finland; ^2^FinVector Vision Therapies Oy, 70210 Kuopio, Finland; ^3^FKD Therapies Oy, 70210 Kuopio, Finland; ^4^Department of Medicine, University of Eastern Finland, 70120 Kuopio, Finland; ^5^Gene Therapy Unit, Kuopio University Hospital, 70120 Kuopio, Finland

## Abstract

Rare-cutting endonucleases, such as the I-PpoI, can be used for the induction of double strand breaks (DSBs) in genome editing and targeted integration based on homologous recombination. For therapeutic approaches, the specificity and the pattern of off-target effects are of high importance in these techniques. For its applications, the endonuclease needs to be transported into the target cell nucleus, where the mechanism of transport may affect its function. Here, we have studied the lentiviral protein transduction of the integrase (IN)-PpoI fusion protein using the *cis*-packaging method. In genome-wide interaction studies, IN-fusion proteins were verified to bind their target sequence containing 28S ribosomal RNA (rRNA) genes with a 100-fold enrichment, despite the well-documented behavior of IN to be tethered into various genomic areas by host-cell factors. In addition, to estimate the applicability of the method, DSB-induced cytotoxic effects with different vector endonuclease configurations were studied in a panel of cells. Varying the amount and activity of endonuclease enabled the adjustment of ratio between the induced DSBs and transported DNA. In cell studies, certain cancerous cell lines were especially prone to DSBs in rRNA genes, which led us to test the protein transduction in a tumour environment in an *in vivo* study. In summary, the results highlight the potential of lentiviral vectors (LVVs) for the nuclear delivery of endonucleases.

## 1. Introduction

Delivery of proteins instead of their cDNAs into target cells is a feasible option in certain therapeutic and experimental approaches where the sustained presence of heterologous proteins is not required or desired. Protein transduction is traditionally achieved by the use of polypeptides or protein transduction domains fused to the protein of interest. There are some commonly used tools for such strategies, for example, the human immunodeficiency virus-1 (HIV-1) Tat protein, the* Drosophila melanogaster* antennapedia peptide (Ant), and the VP22 protein of the herpes simplex virus. Although these approaches are efficient* in vitro*, they suffer from the lack of appropriate vectorization, which hampers efficient protein delivery into specific tissues, cell types, or distinct cell compartments [1–4]. LVVs and vector derived virus-like particles (VLPs) have been used to transport therapeutic proteins into targeted cells as HIV-1 Vpr fusion proteins [5]. This so-called* trans*-packaging strategy [6] has been used in most studies where proteins have been packaged into lentiviral VLPs and LVVs. The cDNA of a foreign protein is cloned in-frame to vpr-gene in a separate expression plasmid, and the Vpr-fusion protein becomes incorporated into the newly formed vectors and VLPs through its interaction with the p6 protein of the Gag. However, the utility of the* trans*-packaging method may be subject to limitations in certain applications owing to the proapoptotic and cytotoxic properties of Vpr [7].

We have previously described a* cis*-packaging method, which is based on generating fusion proteins with the HIV-1 IN, the protein responsible for transgene integration [8]. Proteins of interest are cloned to the C-terminus of IN in the* pol*-gene of the vector packaging plasmid. Pol becomes translated as a Gag-Pol precursor polyprotein through a ribosomal frameshift that occurs at a frequency of 5–10% [9]. In addition to IN,* pol* contains the genes for the viral enzymes reverse transcriptase (RT) and protease (PR), the latter being responsible for the timely order of precursor protein processing that leads to virion maturation. Processing of the Pol to its subunits occurs after virion budding. IN-fusion proteins thus become incorporated into new vector particles as a part of the large Gag-Pol polyprotein, and they are released from Pol only after the vector particle has left the producer cell, thereby enabling the packaging of both toxic and inert proteins into LVVs and VLPs. Such particles are devoid of Vpr and retain their transgene-transferring capability and integration proficiency, with the latter property depending on the vector-contained IN-protein composition [8, 10].

I-PpoI is a homing endonuclease from the slime mold* Physarum polycephalum* [11]. It has a 15 bp cleavage site present in the 28S ribosomal RNA (rRNA) gene, which is highly conserved throughout the eukarya. The rRNA genes are found as hundreds of copies in the ribosomal DNA (rDNA) areas of the short arms of the acrocentric chromosomes 13, 14, 15, 21, and 22 [12–14]. In addition to numerous rDNA sites, I-PpoI recognition sites can be found also elsewhere in the genome.

In this work, we have characterized the genomic DNA-binding specificity of the IN-I-PpoI fusion protein after its delivery into cells by LVVs. It is well-documented that IN takes advantage of cellular transcription factor LEDGF/p75 in order to gain access to chromatin [15, 16]. Therefore characterizing the specificity of IN-I-PpoI's target binding is important for the applicability of* cis*-packaging. In addition to binding specificity, we wanted to characterize the cytotoxicity of IN-I-PpoI protein transduction. For any nuclease used, extensive amount of genomic DSBs in target sites and off-target activity elsewhere in the genome leads to cytotoxicity. To be able to work with tolerable level of DSBs, for example in homologous-recombination based strategies one needs to adjust the ratio between the endonuclease-induced DSBs and transported proviral DNA. To achieve this, we used an IN-I-PpoI derivative with the H78A mutation in the I-PpoI sequence. This protein was generated to decrease I-PpoI's full enzymatic activity. Unlike the noncutting N119A mutant described before, H78A exhibits reduced catalytic activity [17]. The results confirm that the lentiviral delivery with* cis*-packaging method* per se* is not a determinant of endonuclease protein transduction cytotoxicity but that the characteristics of both the used endonuclease and the target cells play important roles.

## 2. Results

We wanted to ask the following research questions: to what extent the endonuclease binding into host cell DNA is specific, what the amount of off-target interactions is, how cells do respond to different levels of I-PpoI-derived DSBs, and whether the DNA-cleavage can be used successfully in totally different application—in an* in vivo* tumour model. For the experiments, vector batches containing either IN-I-PpoI or IN-I-PpoI_H78A_ were produced (Table 1) and pooled where needed. Correct incorporation of the new IN-I-PpoI_H78A_ fusion protein into LVVs and its activity were verified by immunoblotting using an antibody against HIV-1 IN (see Supplementary Figure S1A-C in Supplementary Material available online at http://dx.doi.org/10.1155/2014/379340). Mixed multimer or* trans*-complemented vectors were generated by mixing equal amounts of the fusion protein-containing and either wild-type (wt) IN or inactive IN_D64V_-coding packaging plasmids. The H78A mutation was introduced into I-PpoI in the fusion-IN to investigate the effects of a less active endonuclease. Replacing the histidine in the catalytic site of I-PpoI with an alanine has previously been reported to decrease the enzyme's activity to 48% of wild-type level [17, 18].

### 2.1. ChIP-Analysis Confirms That I-PpoI Is Capable of Undergoing an Interaction with Its Target Sequence

**Figure 1:**
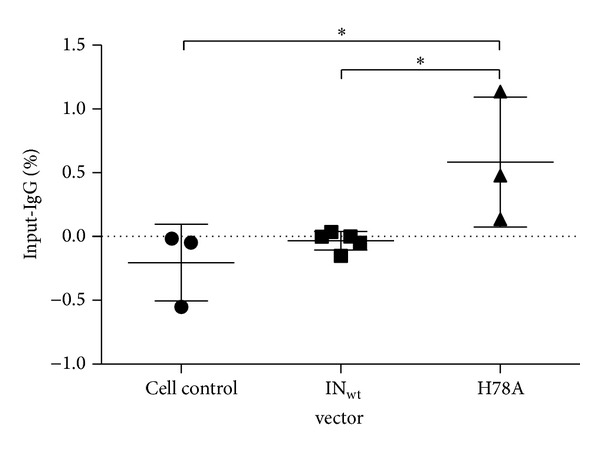
Interaction of different IN proteins with the I-PpoI site on chromosome 1. The binding of IN-I-PpoI_H78A_ proteins (H78A in short) with the I-PpoI target site in 1p32.2 was studied using ChIP. The results (mean ± SD) represent measurements from two independent ChIP experiments. Samples were derived from three independent and nonsimultaneous transductions. No interaction with target sequence was observed in the wt IN samples studied. Results were analyzed with one-way ANOVA and Bonferroni's multiple comparison test. ***P < 0.001; **P = 0.001 to *P* < 0.01; *P = 0.01 to *P* < 0.05.

Before the large-scale studies, the interaction of IN-I-PpoI_H78A_-containing vectors with a single I-PpoI target site on chromosome 1 (1p32.2) was confirmed using chromatin immunoprecipitation (ChIP) analysis. A statistically significant interaction between the vector-carried proteins and the target site was observed, whereas no positive qPCR signals were detected from nontransduced or LVV IN_wt_ transduced cells (Figure 1).

After verifying the target sequence binding by IN-I-PpoI_H78A_, the IN-endonucleases' chromatin interactions were studied using ChIP-sequencing, which allows mapping of all protein contacts with cellular DNA. Studies were conducted using the same vectors as used in the cytotoxicity study comprising HeLa cells and MRC-5s: the* trans*-complemented LVVs IN_wt_+IN-I-PpoI_H78A_ and IN_D64V_+IN-I-PpoI_H78A_. Since the rDNA is not included in the chromosomal DNA sequences of the latest human genome version GRCh37/Hg19, hits in the unplaced contig ChrUn_gl000220 were counted and regarded as rDNA interactions [10]. The unplaced contig contains one full-length and one partial rDNA repeat, in addition to an unknown area to which no rRNA gene sequences could be mapped.

**Figure 2:**
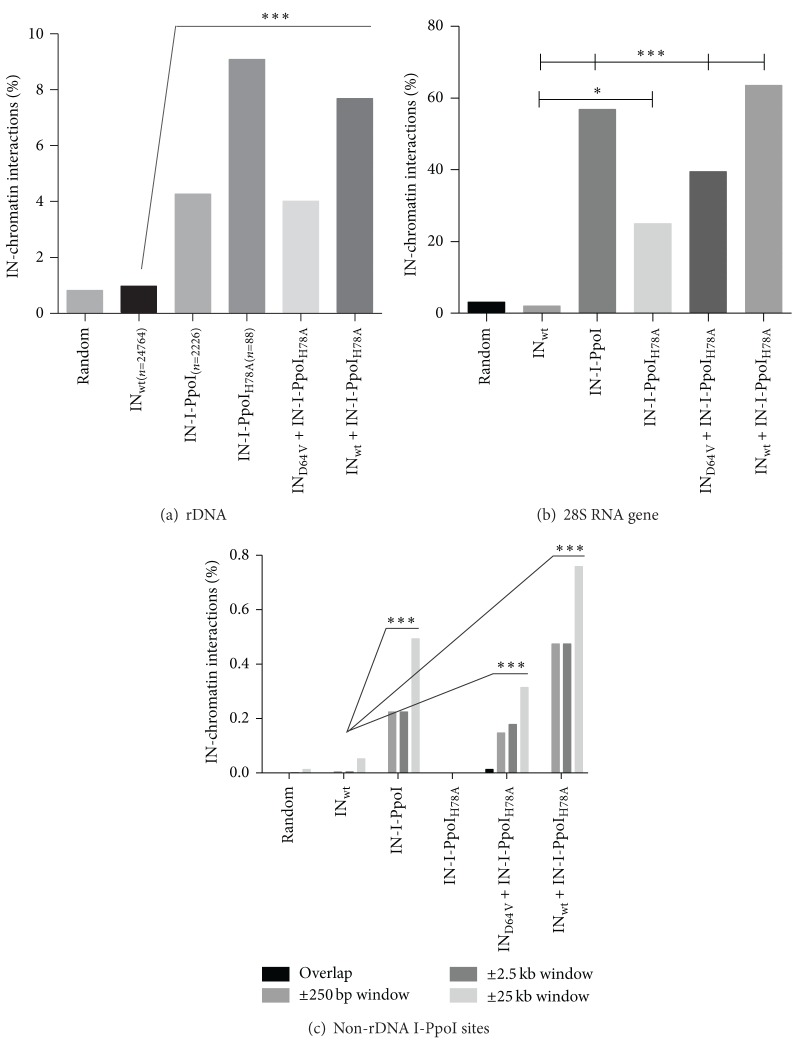
ChIP-sequencing-mapped IN-chromatin interactions at (a) the rDNA repeat area, (b) within the 28S rRNA gene, and (c) in the non-rDNA-localized genomic I-PpoI sites (±different window sizes around the I-PpoI site). In (a) and (c), the results are shown as a percentage of *n*, where *n* indicates the numbers of unique interactions aligning to the genome version GRCh37/Hg19. In (b), the results are shown as a percentage of all interactions aligning to the ChrUn_gl000220. “Random” represents the theoretical probability of an interaction occurring in one of the ~600 copies 43 kb rDNA repeats, in 28S RNA inside ChrUn_gl000220, or into any of the 15 bp I-PpoI recognition sites with window sizes of ±250 bp, 2.5 kb, or 25 kb surrounding the I-PpoI site. Statistical significances are calculated using the chi-square ((a) and (c)) and Fisher's exact test ((b)). Statistical analyses are conducted against wt IN (random value is not included in statistical analyses; shown for illustrative purposes only). In (a), the differences between wt IN and all the IN-I-PpoI-containing groups are significant (*P* < 0.001). The situation is the same in (b) and (c) (*P* < 0.0001), except for the IN-I-PpoI_H78A_, which has a *P* value of 0.0174 in (b) and zero interactions localizing within the non-rDNA I-PpoI site (±0 bp) in (c). ***P < 0.001; **P = 0.001 to *P* < 0.01; *P = 0.01 to *P* < 0.05.

All of the studied LVVs carrying IN-fusion proteins exhibited an increased interaction with the rDNA repeat, when compared to the nonmodified LVV IN_wt_ (Figure 2(a)). Inside the repeat, interactions occurring in the 28S rRNA gene were further studied, since this structure harbors the 15-bp I-PpoI target site (Figure 2(b), % of all rDNA interactions). After transduction with fusion protein containing LVVs, on average, the majority (~52%) of the interaction sites within rDNA were localized to the 28S rRNA (2.30% of total interactions). In the case of IN_wt_ control, only 2.1% of rDNA interactions took place in the 28S rRNA (0.02% of total interactions). Instead of 28S rRNA, IN_wt_ interactions were mostly mapped to the intergenic spacer area (IGS, ~41% of rDNA-localizing sites). In addition to rDNA, the interaction of different LVV-carried IN-proteins with non-rDNA I-PpoI sites in the genome (Figure 2(c)) was studied using four window sizes: (i) ±0 bp (within the 15 bp site), (ii) ±250 bp, (iii) ±2.5 kb, and (iv) ±25 kb. With the exception of the IN-I-PpoI_H78A_, which had only 88 final genomic interaction sites aligned, all IN-fusion proteins displayed enhanced interaction with the I-PpoI target sites when compared to the IN_wt_ at window sizes ±250 bp, ±2.5 kb, and ±25 (*P* < 0.0001; Figure 2(c)). Taken together, it is concluded that the differences in cytotoxicity between the IN-I-PpoI with native endonuclease activity and its mutated form IN-I-PpoI_H78A_ are not due to a reduced target DNA-binding ability of the latter, as verified by ChIP sequencing.

### 2.2. The Cytotoxicity of IN-I-PpoI Protein Transduction Is Dependent on the Enzyme's Activity and Amount of Protein Packaged into Lentiviral Particles

**Figure 3:**
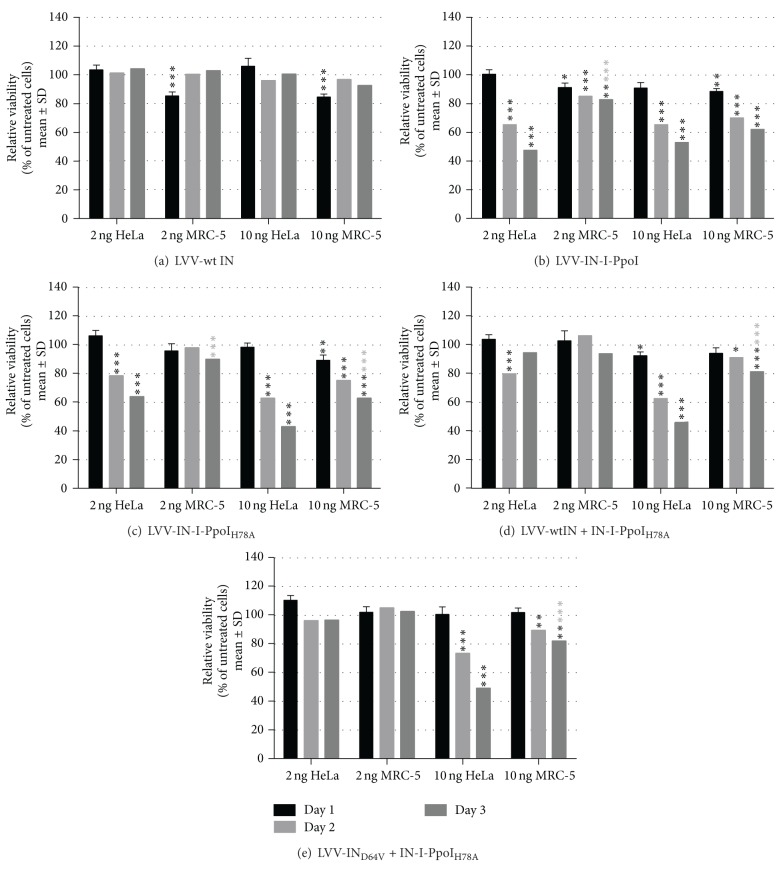
The effects of IN-I-PpoI and IN-I-PpoI_H78A_ protein transduction on the viability of HeLa and MRC-5 cells. Cells were transduced with 2 or 10 ng of p24 per well, and their viability was followed for three days. Significant negative deviations from untreated cells (drop in viability) are shown with black asterisks and significant differences between the cell lines at day three with gray asterisks (one-way ANOVA followed by Dunnett's multiple comparison test). ***P < 0.001; **P = 0.001 to *P* < 0.01; *P = 0.01 to *P* < 0.05.

I-PpoI has at least eight perfect full-length recognition sites in the human genome [10] in addition to approximately 400–600 sites found in the rDNA [19]. The high number of potential cleavage sites poses a remarkable challenge to the DNA damage repair capabilities of the transduced cells. An excess of endonucleases can result in genomic instability and genotoxicity, as observed with zinc finger nucleases [20–22]. We wanted to reduce the amount of induced DSBs by modulating the cleavage activity (H78A mutant) and vector endonuclease content (mixed multimers) without reducing the amount of viral particles, which would have meant reducing the number of transgenes per cell. HeLa and MRC-5 cells were transduced with the vectors carrying either of the IN-endonuclease proteins using LVV IN_wt_ vectors as a control. The viabilities of the vector-treated and nontransduced cells were compared during days one to three after transduction (Figures 3(a)–3(d), black asterisks). As expected, the fully active I-PpoI decreased the viability of transduced cells (Figure 3(b)). Transduction with the lower endonuclease activity-containing LVV IN-I-PpoI_H78A_ also decreased the viability of HeLa cells even at the 2 ng p24 dose, whereas MRC-5 cells remained unaffected by this concentration (Figure 3(c)). The mixed multimer vectors LVV IN_wt_/IN_D64V_+IN-I-PpoI_H78A_ did not cause any permanent reduction in the viability of either tested cell lines with the 2 ng p24 vector dose used (Figures 3(d) and 3(e)). With the 10 ng dose, a cytotoxic effect was observed, confirming that the endonuclease was still packaged into the vectors. As expected, reducing the content and activity of the IN-endonuclease in vector particles proved to be a feasible way of modulating the cytotoxicity. In addition, the characteristics of the target cells affect the cytotoxicity encountered with this approach, the* cis-*packaging method itself being well suited for nuclear delivery of the endonucleases. Differences in cytotoxicity between vectors carrying the IN-endonuclease proteins can result from their different abilities to recognize I-PpoI sites, resulting in off-target effects. The ability of the IN-I-PpoI forms to bind to their genomic target sites was therefore next studied using chromatin ChIP-techniques.

### 2.3. Cell Culture Studies Indicate Increased Cytotoxicity in Tumour-Derived Cell Lines

**Figure 4:**
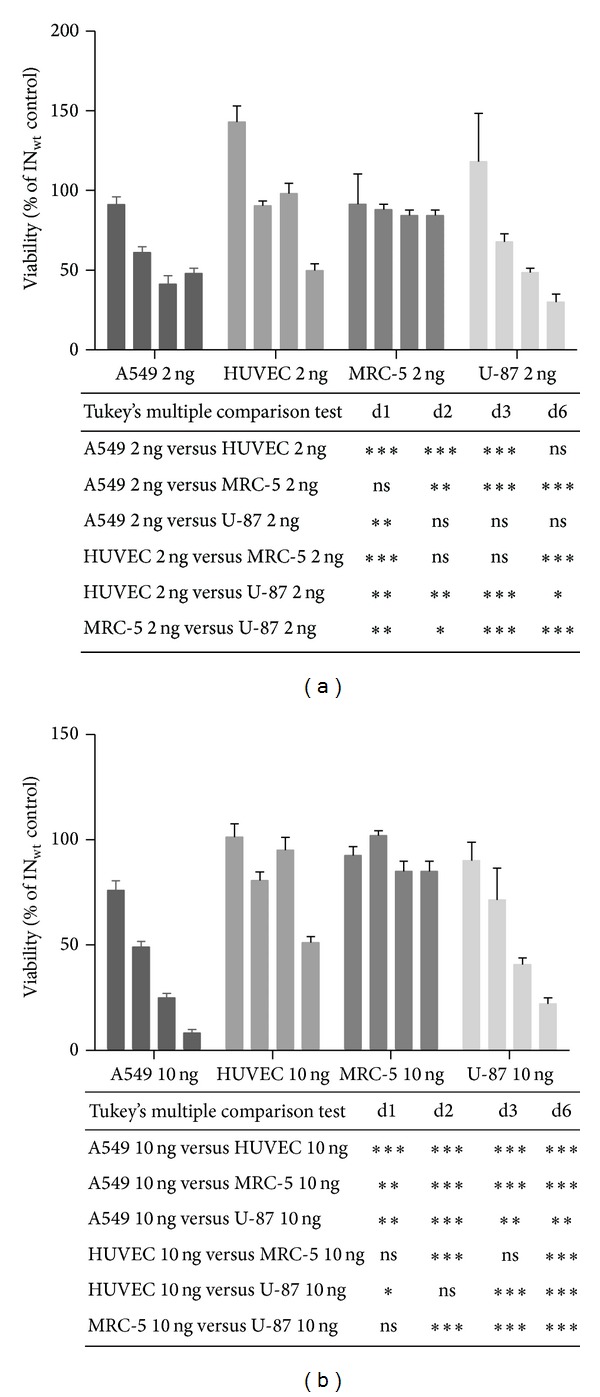
A cytotoxicity study in different cell lines using two concentrations of IN-I-PpoI. Cell viabilities measured by a luminescent cell viability assay are shown as percentages of the wt IN control vector-transduced cells (mean ± SD) after a vector dose of 2 ng (a) and a vector dose of 10 ng (b) of p24. The bars represent viability after LVV mediated I-PpoI protein transduction at different time points post transduction (1, 2, 3 and 6 days, from left to right). Results were analyzed with two-way ANOVA and the Tukey's multiple comparisons test. ***P < 0.001; **P = 0.001 to *P* < 0.01; *P = 0.01 to *P* < 0.05.

Although excessive DNA double strand break (DSB) formation is cytotoxic, site-specific cleavage can be exploited for therapeutic purposes in the form of genome editing and gene insertion through enhanced homologous recombination (HR [23, 24]). To characterize whether cancerous cells in addition to the tested HeLa cells would be more sensitive to IN-I-PpoI-originated cytotoxicity, we conducted a viability study in a panel of cells: 293T, A549, ARPE-19, BT4C, HeLa, HepG2, HUVEC, MRC-5, U-87, and U-255 cells (see Materials and Methods for descriptions). Cells were transduced with different concentrations of the vector ranging from 2 to 10 ng of p24 per well, which had been assessed previously to be non-cytotoxic using the LVV-wt IN (Figure S2). The active endonuclease-containing LVV IN-I-PpoI caused a significant reduction in the viability of most cell lines, when compared to untreated cells (Figure 4 and supplementary Figure S3). The cell line responses to LVV IN-I-PpoI transduction varied considerably. Generally, when compared to cancer cell lines, the nontumorigenic and nontransformed MRC-5, ARPE-19, and primary HUVEC cells exhibited slightly less extensive reductions in viability after LVV-IN-I-PpoI vector treatment at the end of the study (Figures S3 and S4), although the impact on HUVEC cells was difficult to interpret due to the deviant viability at the last time point. However, overall differences between the cell lines were moderate. With a high LVV dose, especially, the A549 and BT4C cells, which likely also suffer from cancer-specific defects in their DSB repair pathways, perished or stopped dividing. Similarly, 293T cells, despite not being a cancer cell line, exhibited extensive cytotoxicity in response to LVV IN-I-PpoI transduction. HEK293-derived cell lines, such as 293T, are not suitable models for healthy cells in DSB experiments, since they are known to express the adenoviral oncoprotein E1B55K, which disrupts the functionality of the DNA damage response pathways [25].

### 2.4. IN-I-PpoI Protein Transduction Reduces Tumour Growth in a Subcutaneous Tumour Model in Mice

After observing a difference in cell death and/or growth arrest between tumour and normal cell lines* in vitro*, experiments with solid tumours were initiated to determine whether LVV IN-I-PpoI protein transduction could promote a similar effect* in vivo*. Two types of IN_wt_ vectors were used as controls: one carrying the GFP-transgene for determination of transduction efficiency and the other containing the well-characterized thymidine kinase (TK) transgene, which has antitumorigenic effects in cancer cells when combined with ganciclovir injections [26, 27]. Tumours were induced by the transplantation of A549 cells into the flanks of nude mice and then the tumours were injected with vectors 8 to 14 days after implantation. The development of tumour size was analyzed until 22 days after transduction.

**Figure 5:**
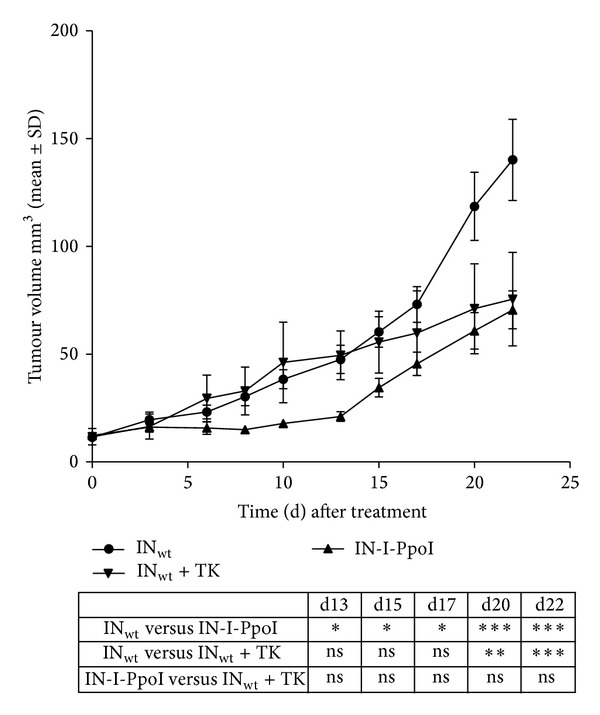
Development of tumour volumes following LVV transduction. Volume changes (mean ± SD) of subcutaneous A549 tumours treated with wild-type control vector (wt IN), active endonuclease containing vector (IN-I-PpoI, I-PpoI in short), or thymidine kinase transgene-containing vector (wt IN+TK) are shown. Group sizes were LVV IN_wt_, *n* = 17; LVV IN-I-PpoI, *n* = 18; and LVV IN_wt_ TK, *n* = 6. Differences between groups were analyzed by two-way ANOVA and Tukey's multiple comparisons test. ***P < 0.001; **P = 0.001 to *P* < 0.01; *P = 0.01 to *P* < 0.05.

**Figure 6:**
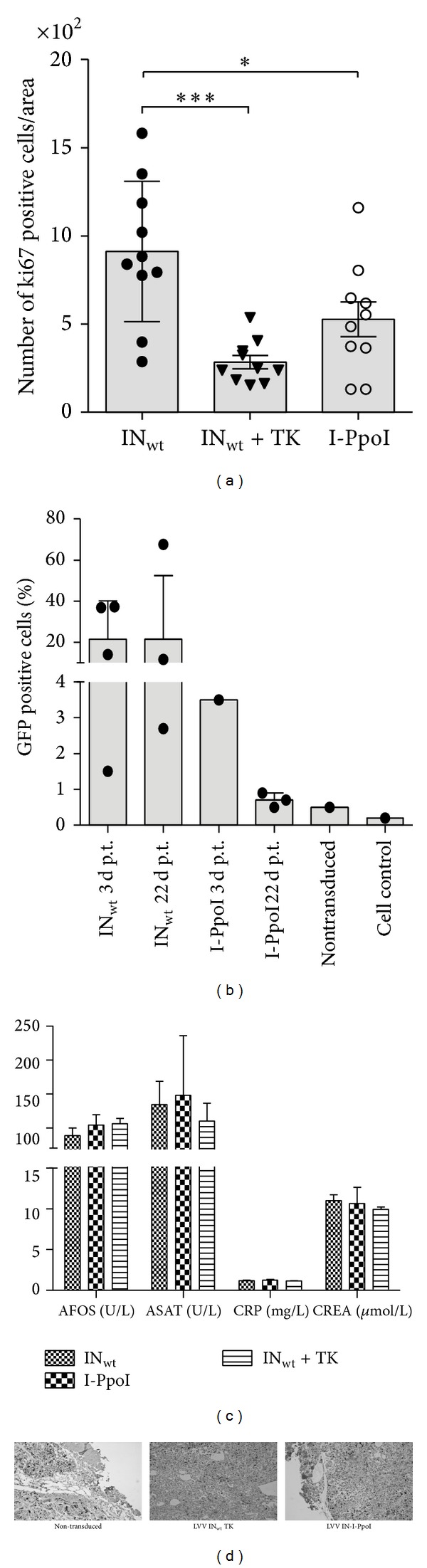
Tumour and blood sample analysis. The amount of proliferating cells per area is shown in (a) (mean ± SD) and examples of stained tumour histological samples in (d). GFP expression (mean ± SD) in dissociated tumour cells is presented in (b) as measured by flow cytometry analysis of dissociated tumour cells or the cultured cell control. The results (mean ± SD) of blood sample analysis from the samples drawn at the end of the* in vivo* experiment are shown in (c) LVV IN_wt_, *n* = 4; LVV IN-I-PpoI, *n* = 5; and LVV IN_wt_ TK, *n* = 3). No statistical differences were found between groups in (b) or (c). CRP: C-reactive protein, CREA: creatinine, ASAT: aspartate aminotransferase, and AFOS: alkaline phosphatase. Statistical analysis: one-way ANOVA in (a) and (b) and two-way ANOVA in (c) combined with Bonferroni's ((a) and (c)) and Tukey's (b) multiple comparisons tests. ***P < 0.001; **P = 0.001 to *P* < 0.01; *P = 0.01 to *P* < 0.05.

Throughout the experiment, tumour sizes were found to remain smaller in the LVV IN-I-PpoI-injected mice than in control groups. The efficiency in tumour size reduction was similar to that obtained with LVV IN_wt_ TK (Figure 5). For the first 13 days, tumour sizes in the IN-I-PpoI group remained stable without any significant changes in tumour volumes. All tested tumours were aggressively proliferating with malignant irregularly shaped cells (Figures 6(a) and 6(d)). Flow cytometry analyses of randomly selected dissociated tumours were performed for the GFP transgene-containing LVV IN_wt_ and LVV IN-I-PpoI treated mice at 3 and 22 days after transduction (Figure 6(b)). Successful transduction and cellular entry of LVVs after the injection procedure were verified with LVVs containing IN_wt_. Probably due to the IN-I-PpoI-carrying vector's low integration activity and its apparent cytotoxicity, at day 22, LVV-IN-I-PpoI treated tumours showed only minimal green fluorescent protein (GFP) expression (0.7–0.9%), being only slightly higher than the baseline value (0.5%) set for nontransduced tumour control. No differences in body weight loss, animal behavior, or signs of inflammation and liver or kidney failure were detected in any of the groups (Figure 6(c)). These results suggest that LVV protein transduction can be successfully used also in more difficult-to-transfect cellular environments.

## 3. Discussion

We have previously shown that LVV IN-I-PpoI can induce targeted DSBs and its cleavage-impaired mutant can increase transgene integration into rDNA [10]. However, there was no direct proof of a protein interaction with or close to the aimed I-PpoI recognition sites. Here, we have addressed this open question by using the ChIP sequencing with IN-I-PpoI and its catalytically impaired version IN-I-PpoI_H78A_. The fusion protein containing vectors exhibited increased chromatin interactions involving rDNA repeats and the 28S rRNA genes within these regions. The result highlights the feasibility of fusing chromatin-interacting proteins to IN. In addition, the non-rDNA-related I-PpoI recognition sites were more frequently present in the IN-fusion protein data sets as compared to control, demonstrating IN-I-PpoI's ability to interact with its target sites also outside rDNA.

The number of IN molecules contained in a lentivirus particle is limited with estimates between 20 and 250 molecules [28, 29]. IN-fusion proteins may be present at lower levels because of potentially inefficient IN-fusion protein expression and packaging into new particles in producer cells. We have not determined the number of particles lacking the IN-fusion protein in our vector preparations. However, since* gag-pol* is transcribed in a fixed relationship to* gag* [9], the stoichiometry between (Gag-)Pol and Gag should be preserved when using the IN-fusion protein-containing packaging plasmids for vector production. Based on a stoichiometry value of 2000 copies of p24 capsid proteins per viral particle, a 10 ng dose should correspond roughly to 10–100 TUs per cell in our experimental settings [30, 31]. Considering the strong effects on cellular viability encountered with low to moderate vector doses, we can conclude that the number of IN-molecules per vector particle does not represent a limiting factor, at least not in the two different study types presented here. This information could be useful for applications such as DSB-enhanced HR, which could benefit from the protein transduction technology we have described. Once inside the cell, the protein can be delivered into the nucleus or potentially to the cytosol, if the step of nuclear import is disabled for the modified vectors. According to our results, IN-I-PpoI LVVs do not cause extensive cytotoxicity in all cell lines when administered at low doses (Figures 1, 4, and S3), despite the fact that I-PpoI has several hundreds of recognition sites in the human genome. About 30% of the genomic I-PpoI sites were recently analyzed to become cleaved after I-PpoI administration [32].

Susceptibility to DSB formation and reduced DNA-repairing capability through ionizing radiation or other forms of cellular stress are present in most cancers. In normal cell lines, the number of DSBs evoked by a certain amount of stress is largely constant and the outcome is mediated by the efficiency of the DSB repair, whereas, in cancer cells, the increased total number of DSBs may present a major challenge for maintaining cellular viability. Apparently, the repair of the number of DSBs caused by LVV IN-I-PpoI transduction does exceed the capacity of certain cells' DSB repair machineries, as we observed high cytotoxicity after LVV IN-I-PpoI transduction in some transformed cells lines* in vitro*. The observed cytotoxicity in cancer cells prompted us to test the effects of IN-I-PpoI fusion protein* in vivo*. We demonstrated successful transduction of cells in a challenging tumour environment with inhibition of tumour growth achieved at a similar level as seen with the well-characterized TK-ganciclovir system.

This and other LVV/VLP-mediated protein transduction applications continue to offer several possibilities in life-sciences, and the broad range of lentiviral pseudotyping possibilities extends the selection of target cells and tissues. Examples of potential applications include the pIPSCs (proteins induced pluripotent stem cells) techniques [33], where proteins with carcinogenic potential have been directly transferred into cells instead of delivering them via viral transduction via viral transduction of their cDNAs. Another example is related to immunotherapies, where cells can be transduced with proteins that initiate immunogenic cascades. The obvious applications are in gene therapy and DSB-enhanced HR strategies, with the possibility of transporting nucleases into target cells without adding Vpr to the vector production system. For HR applications, endonucleases with unique cleavage sites in the human genome would be preferred catalysts for DSB generation. However, it remains to be determined whether the amount of packaged IN-fusion proteins is sufficient for proteins with lower activity than observed for I-PpoI, to exert their specific cellular functions. In summary, by incorporating the DNA-cleaving meganuclease I-PpoI into the 3rd generation LVVs, we showed that LVVs with* cis*-packaged LVVs could be used as versatile tools to transfer genome modifying proteins into target cells.

## 4. Materials and Methods

### 4.1. Plasmids and Vector Production

The packaging plasmid pMDLg/pRRE-IN-I-PPoI_H78A_ was generated from pMDLg/pRRE-IN-I-PpoI [10] with the QuikChangeII XL Site-Directed Mutagenesis Kit (Stratagene) using primers H78A Forw (5′-CCACAGATGGGGATCCGCCACAGTCCCTTTTCTATTAGAACCGG-3′) and H78A Rev (5′-CCGGTTCTAATAGAAAAGGGACTGTGGCGGATCCCCATCTGTGG-3′). Correct packaging of the IN-I-PpoI_H78A_ into LVVs was verified by immunoblotting using an antibody against HIV-1 IN, as described previously [10]. VSV-G pseudotyped third generation LVVs were produced, concentrated, and titered with p24 ELISA and flow cytometry as described [8]. TK transgene-containing vector LVV INwt TK with an unmodified IN-content was generated to serve as a control in the* in vivo* study. With the exception of LVV INwt TK, all vectors carried the GFP transgene under the control of the phosphoglycerate kinase (PGK) promoter. The functionality of IN-I-PpoI_H78A_ to cleave I-PpoI sites was verified by analyzing vector-extracted proteins in a plasmid cleavage assay (Figure S1B) and after LVV protein transduction in MRC-5 cells (Figure S1C). The packaging plasmids used were pMDLg/pRRE, pMDLg/pRRE-IN_D64V_ pMDLg/pRRE-IN-I-PpoI, and pMDLg/pRRE-IN-I-PpoI_H78A_. LVVs containing mixed IN molecule multimers were produced using two different packaging plasmids in equimolar amounts. A 345 bp stretch of genomic DNA around the I-PpoI recognition site in rDNA was amplified from HeLa cells' genomic DNA using the primers rDNA 5′Ppo (5′-GACTTAGAACTGGTGCGGAC-3′) and rDNA 3′Ppo (5′-CACTTATTCTACACCTCTCATG-3′) and inserted into the EcoRV-site of pGEM-T Easy (Promega) to generate the plasmid prEasy used in testing the restriction enzyme activity of LVV and VLP extracted core proteins.

### 4.2. Plasmid Digestion and rDNA Cleavage by LVV- and VLP-Extracted Cores

Crude extracts of vector or VLP preparations were prepared by mixing equal volumes of LVV or VLP preparations (10–60 *μ*L) with a lysis buffer that consisted of 0.5% Igepal CA-630 (Lonza) and one complete protease inhibitor tablet (Roche) in 25 mL of DPBS (Lonza). Vector particles were gently vortexed in the lysis buffer, incubated at room temperature for 5 minutes, and centrifuged at 4°C 14000 rpm for 8 minutes. The pellet was resuspended in DPBS in a volume that was half of the original LVV or VLP volume, except for LVV-IN-I-PpoI_H78A_ which was resuspended into the original volume of 10 *μ*L. Digestion reactions were set up with 500 ng of the plasmid DNA, 1× I-PpoI buffer (Promega), 1× BSA (Promega), and 5 *μ*L or 10 *μ*L of the protein extract in a total volume of 30 *μ*L. Digestions were carried out at 37°C for 90 minutes, after which 2 *μ*L of ScaI and 1× ScaI buffer (Fermentas) were added to the reaction, and the total volume was raised to 50 *μ*L with water. The reaction was incubated at 37°C for an additional hour and analyzed by agarose gel electrophoresis. The positive control digestions with I-PpoI were implemented as above, but, instead of the vector extracts, 1 *μ*L of I-PpoI (Promega) was mixed into 10 *μ*L of DPBS (Lonza). The rDNA cleavage test in transduced cells was performed as previously described [10].

### 4.3. Cell Culture and Transductions

The tested cells and their ATCC (American Type Culture Collection) numbers were 293T (derivatives of the embryonic kidney cell line HEK293, CRL-11268), A549 (epithelial lung carcinoma, CCL-185), ARPE-19 (normal retinal pigment epithelia cell line, CRL-2302), BT4C (rat glioma cell line), HeLa (cervical cancer, CCL-2), HepG2 (hepatocellular carcinoma, HB-8065), MRC-5 (normal lung fibroblast, CCL-171), U-251 (glioblastoma astrocytoma, HTB-17), and U-87 (glioblastoma astrocytoma, HTB-14). HUVECs (normal primary human umbilical vein endothelial cells) were included in the study. 293T, ARPE-19, U-87, and U-251 cells were cultured in Dulbecco's modified Eagle's medium (DMEM; Sigma) supplemented with 10% fetal bovine serum (FBS; Hyclone) and 1% penicillin-streptomycin (P/S). For BT4C cells, also 2 mM GlutaMAX (Invitrogen) was added. A549 and MRC-5 cells were cultured in DMEM supplemented with 1% penicillin-streptomycin (Sigma), 1% nonessential amino acid solution (Sigma), 1% sodium pyruvate solution (Sigma), and 10% FBS at 37°C in a 5% CO_2_-containing humidified atmosphere. HeLa cells were cultured without nonessential amino acid solution and sodium pyruvate. HepG2 cells were cultured in minimum essential medium Eagle with supplements identical to A549 and MRC-5 medium and 2 mM GlutaMAX. HUVEC cells were cultured with EGM endothelial cell growth medium (Lonza) containing EGM SingleQuot kit supplements (Lonza). In the* in vitro* studies, cells were transduced with LVVs by diluting the vector into prewarmed media before adding the mixture to cells.

### 4.4. Cytotoxicity Assay

The cytotoxicity of LVV IN-I-PpoI was tested on several cell lines using the CellTiter-Glo Luminescent Cell Viability Assay (Promega). The unmodified IN_wt_ was used as a control. On the day before transduction, 10000 MRC-5 cells and 4000 or 5000 A549 cells were seeded onto 96-well microplates (B&W Isoplate-96 TC, Perkin Elmer). Cells were transduced with one to four different LVV dilutions to load cells with 2, 5, 10, or 100 ng of p24 per well. For all cell lines, at least, the 10 ng transduction was conducted. Cytotoxicity of the vector treatments was assayed by adding the CellTiter-Glo reagent and reading the luminescence with Fluoroskan Ascent FL plate-reader (Thermo Fisher Scientific) at time points 1, 2, and/or 3 and 6 days after transduction.

### 4.5. ChIP

ChIP for detecting IN chromatin interaction was performed using antibodies to HIV-1 IN amino acids 23–34 and 142–153 (cat. nos. 757 and 3514; AIDS Research and Reference Reagent Program, Division of AIDS, National Institute of Allergy and Infectious Diseases (NIAID), National Institutes of Health (NIH)). Cells on 10 cm plates were transduced with a target value of MOI 5–10 and incubated for ~6–7.5 h (for most samples, transduction was made in 2-3 steps with at 0.5 hour intervals) before crosslinking with 1% formaldehyde for 15 min. Cells were lysed and DNA was sonicated into ~500 bp fragments and precleared with salmon sperm DNA/protein A agarose slurry. Immunocomplexes were collected with antibody and Magna ChIP protein A magnetic beads (Millipore). DNA was released from immunocomplexes by proteinase digestion. For detection of interactions near to the target sites, primers 3′ Chr1 GATCTCACTCAACCCACCACG and 5′ Chr1 GGGACACTTCACAGCACTCTCC were used in qPCR reaction (95°C 10′, [95°C 30′′, 64°C 30′′, 72°C 30′′] × 50, 72°C 10′) with master mix Maxima SYBR Green/Rox qPCR Master (Fermentas). In the ChIP sequencing of the samples, DNA end polishing and 3′ dA addition were performed before ligation of barcoded adapters which allow simultaneous sequencing of samples from multiple origins. After the ligation, DNA fragments of size ~200–350 bp were gel-extracted from 2% agarose gel. Fragments were amplified by PCR (98°C 30′′, [98°C 10′′, 65°C 30′′, 72°C 20′′] × 14, 72°C 5′) and gel extraction was repeated. Library was sequenced using Solexa technology (Illumina) at EMBL GeneCore Genomics Core Facility at Heidelberg, Germany. Sequence quality was analyzed using FastQC 0.51 to determine the necessary low quality sequence removal from the 3′ end. Sequences were trimmed according to the Galaxy FastQC results and aligned to the human genomic assembly GRCh37/hg19 (February, 2009) using Bowtie 1.1.2 tool [34]. Unique genomic coordinates were compensated for the 3′ end trimming and analyzed for the overlap with target features.

### 4.6. *In Vivo* Experiments

For subcutaneous tumours, A549 cells were grown to 80% confluency on 15 cm plates, detached with TrypLE express (Invitrogen), and resuspended in Opti-MEM to a concentration of 1 × 10^6^ cells in 50 *μ*L. Resuspended cells were injected into both flanks on the back of male NMRI nude mice (25–37 g, *n* = 26) from Taconic Farms Inc. Before the experimental procedures, animals were allowed to acclimatize for a week. Then, 8 or 14 days after implantation of the tumour cells, mice eligible for experiment (1 or 2 tumours, tumour size of at least 1.75 × 1.75 × 1 mm and maximum volume of 30 mm^3^) were randomly divided into experimental groups and injected with viral vectors as described. Tumours outside these limits were left untreated and their volume was calculated only for purposes of humane endpoints. Vectors were injected in 20 *μ*L of PBS into the center of the tumour with a 25 *μ*L Hamilton syringe (Hamilton, Switzerland) using a 27-gauge needle. The needle was left in place for 1 min after injection. After the injections, the animals were followed for 22 days and tumour volumes were measured three times each week. Five days after injection, TK-treated group received 50 mg/kg ganciclovir (Roche) divided into two i.p. doses per day for 14 days, and mice from the control group wt IN received 0.9% NaCl at a similar schedule. Blood samples were taken at the end of the experiment and serum was extracted with SST microtainer tubes (BD Biosciences). All animal studies had the approval of Experimental Animal Committee of the University of Eastern Finland.

### 4.7. Flow Cytometric Expression Analysis

GFP transgene expression analysis from dissociated tumour cells was performed on days 3 and 22 after vector injections. After animals were sacrificed, randomized tumours were cut out and stored in Opti-MEM until processed. Tumours were placed on cell culture plates containing 3 mL of hyaluronidase, 1 mL of dispase, and 0.02% U of DNase I type IV (Sigma) and minced with a scalpel. Plates were then incubated on shaker at room temperature for 3 h. After incubation, the liquid was filtered through Miltenyi prefiltration columns (30 *μ*m) and fixed with 4% PFA-PBS for 15 min. Samples were analyzed with the BD FACS Canto II and FACS Diva software (BD Biosciences).

### 4.8. Statistics

Unpaired* t*-test with Welch's correction was used to compare IN_wt_ and fusion IN containing LVV titers. ChIP data was analyzed using one-way ANOVA and Bonferroni's multiple comparison test. The* in vitro* and* in vivo* cytotoxicity data was analyzed using one two-way ANOVA and Tukey's multiple comparisons test or the one-way ANOVA followed by Dunnett's multiple comparison test. Statistical analyses for Ki67 positive cells were made with one-way ANOVA combined with Bonferroni's multiple comparisons tests. Tukey's multiple comparisons tests were performed for GFP expression. Blood samples were analyzed with two-way ANOVA Bonferroni's multiple comparisons test. Asterisks are used in figures as follows: ***P < 0.001; **P = 0.001 to *P* < 0.01; and *P = 0.01 to *P* < 0.05. All analyses were performed using GraphPad Prism version 5.01 for Windows, GraphPad Software, http://www.graphpad.com/.

## Supplementary Material

The supplementary material includes following data: The correct vector-incorporation of IN-I-PpoI_H78A_ and its endonuclease activity, verification of non-cytotoxic LVV dose for MRC-5 cells, viabilities of cells treated with varying amounts of LVV-IN-I-PpoI and comparison of the end-viabilities between cancerous and non-cancerous cells.

## Figures and Tables

**Table 1:** Summary of produced vectors.

LVV	*n*	p24 pg/mL	SD p24	FACS (TU/mL)	SD FACS
IN_wt_	5	2.16*E* + 08	1.46*E* + 08	7.45*E* + 09	4.15*E* + 09
IN-I-PpoI	4	3.74*E* + 08	1.36*E* + 08	5.44*E* + 07	5.31*E* + 07
IN-I-PpoI_H78A_	1	1.31*E* + 08	ND	1.89*E* + 08	ND
IN_D64V_+IN-I-PpoI_H78A_	2	6.15*E* + 07	2.71*E* + 07	2.12*E* + 06	2.72*E* + 06
IN_wt_+IN-I-PpoI_H78A_	1	1.00*E* + 08	ND	1.82*E* + 09	ND
IN_wt_ TK	1	2.07*E* + 08	ND	ND	ND

SD: standard deviation; ND: not determined.
