# Supplementary material for: Lentiviral Protein Transduction with Genome-Modifying HIV-1 Integrase-I-PpoI Fusion Proteins: Studies on Specificity and Cytotoxicity
Source: Biomed Res Int. 2014 Apr 22;2014:379340. doi: 10.1155/2014/379340 (PMC4016911; doi:10.1155/2014/379340)
Supplement: Supplementary file 1 — The supplementary material includes following data: The correct vector-incorporation of IN-I-PpoIH78A and its endonuclease activity, verification of non-cytotoxic LVV dose for MRC-5 cells, viabilities of cells treated with varying amounts of LVV-IN-I-PpoI and comparison of the end-viabilities between cancerous and non-cancerous cells. [file 379340.f1.pdf]

Supplementary information

Lentiviral protein transduction with genome-modifying HIV-1 integrase-I-PpoI fusion proteins: Studies on specificity and cytotoxicity

Vesa Turkki[1,5,†], Diana Schenkwein[1,†], Oskari Timonen[1], Tiia Husso[1], Hanna P Lesch[1,4] and Seppo Ylä-Herttuala[1-3,*]

[1]Department of Biotechnology and Molecular Medicine, A.I. Virtanen Institute for Molecular Sciences, University of Eastern Finland, Kuopio, 70211, Finland; [2]Department of Medicine, University of Eastern Finland, Kuopio, 70120, Finland; [3]Gene Therapy Unit, Kuopio University Hospital, Kuopio, 70120, Finland; [4]FKD Therapies Oy, Kuopio, 70210, Finland; [5]FinVector Vision Therapies Oy, Kuopio, 70210, Finland. [†]These authors contributed equally to this work.

[*]To whom correspondence should be addressed. Tel: +358 40 355 2075; Fax: +358 17 163 751; Email: seppo.ylaherttuala@uef.fi

Supplementary information

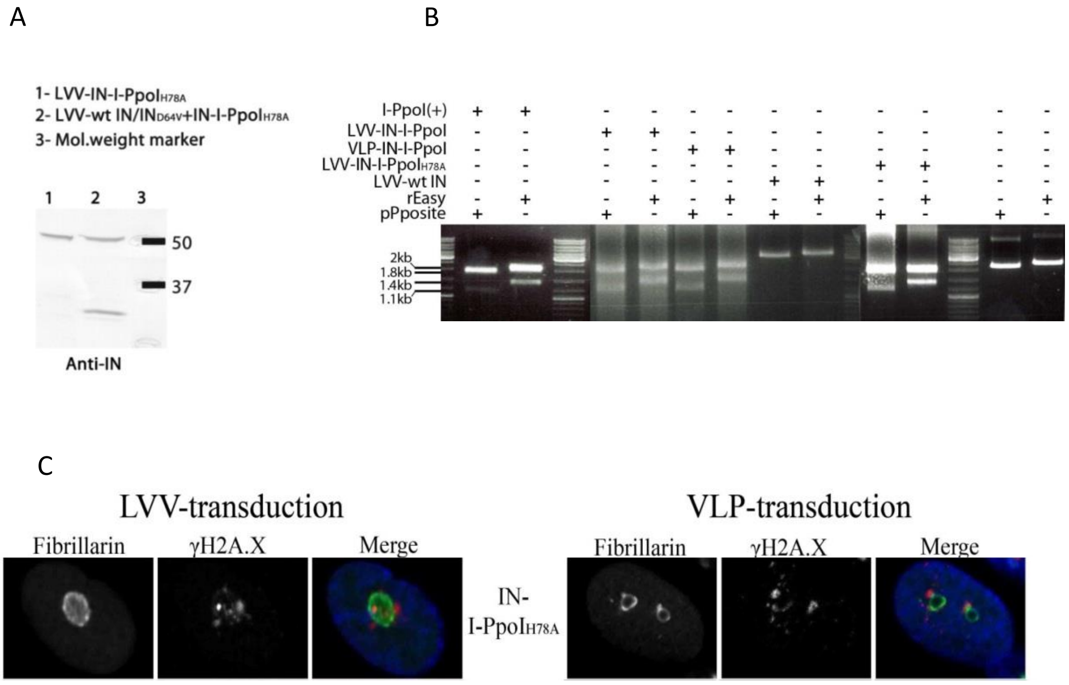

Figure S1. Correct vector-incorporation of IN-I-PpoI$_{H78A}$ and testing its endonuclease activity. (A) The LVVs containing only the fusion protein or a mixture of the fusion protein with IN$_{wt}$ and IN$_{D64V}$ were analyzed by immunoblotting using the antibody against HIV-1 IN. The sizes of molecular weight markers are shown on the right (kDa). Expected molecular sizes: IN-I-PpoI:50 kDa and wt IN and IN$_{D64V}$: 32 kDa. (B) ScaI-linearized plasmids containing single I-PpoI sites (pPposite and rEASY) were digested with LVV- and VLP-extracted viral cores. LVVs IN$_{wt}$ and IN-I-PpoI were used as controls. Positive control (two first lanes): commercial I-PpoI enzyme; negative control (two right-most lanes) linearized plasmids only. Expected sizes of the digestion products are shown on the left. (C) Verifying correct DSB induction at the nucleolar I-PpoI site by immunocytochemistry. An antibody to fibrillarin was used to detect nucleoli (green), an antibody to γH2AX to detect DSB sites (red) and DAPI to visualize nuclei (blue).

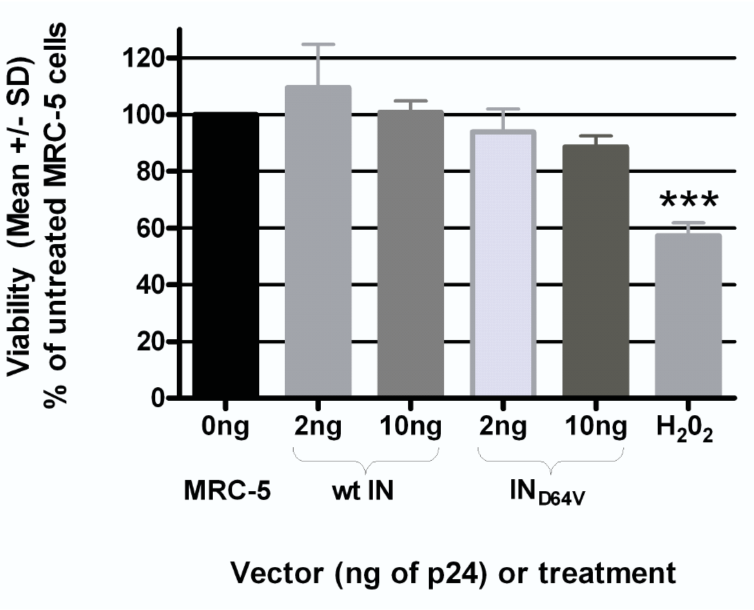

Figure S2: Testing cytotoxicity of vector doses on transduced MRC-5 cells (mean ± SD). The two vector doses used to test the cytotoxicity of IN-fusion protein containing vectors were verified to be nontoxic using the control vectors LVV IN_{wt} and inactive IN containing LVV IN_{D64V} on MRC-5 cells. Hydrogen peroxide was used as a positive control for cytotoxicity. Viability on day 3 post transduction is reported in comparison to the untreated cells (percent of the untreated cells luminescence values) using averages of quadruple well measurements. The only significant difference from the control cells was caused by the hydrogen peroxide treatment positive control (***, $p<0.001$; One-way Anova followed by Dunnett's multiple comparison test).

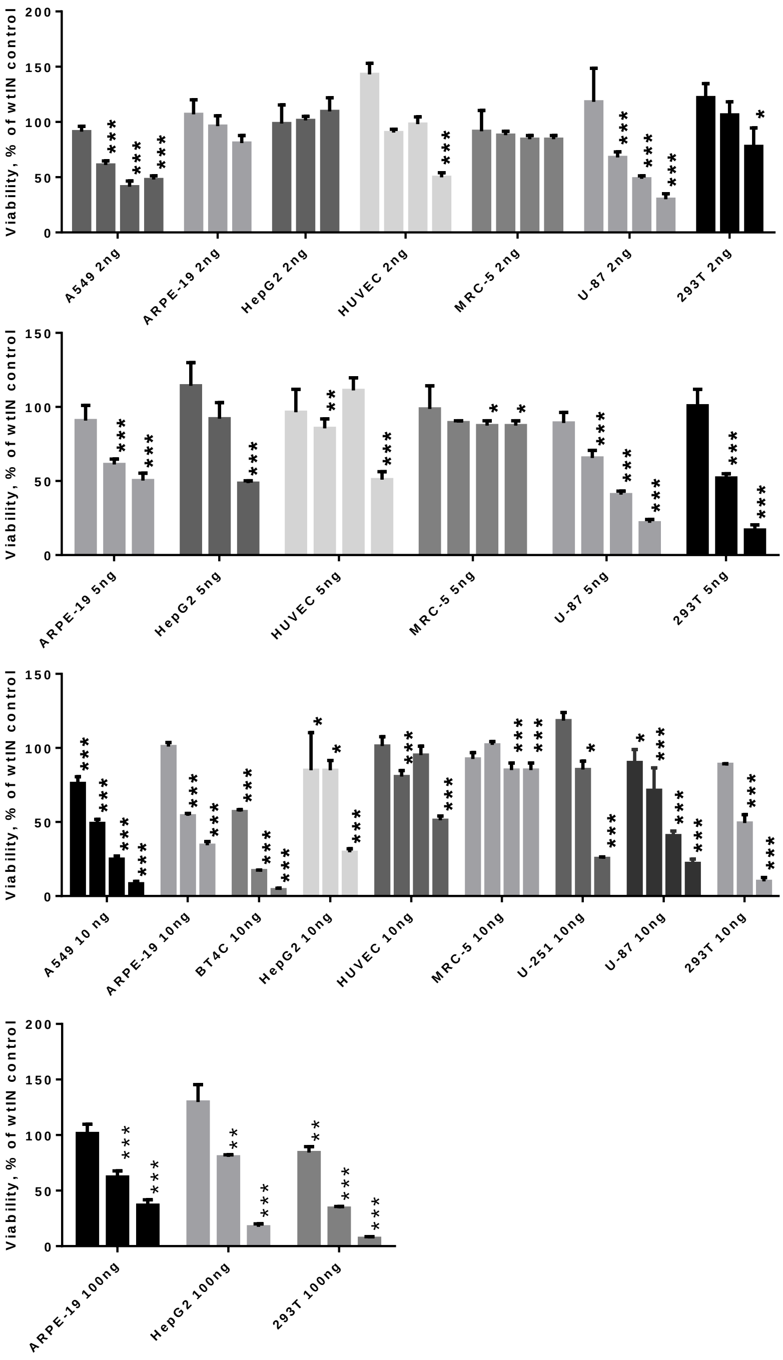

Figure S3: Viabilities of cells treated with varying amounts of LVV-IN-I-PpoI vectors (mean ± SD). Viability at 1, 2, (3) and 6 days post transduction is reported as percentages of the IN_{wt} control vector transduced cells (percent of the LVV IN_{wt} transduced vector luminescence values) using averages from 3-6 wells. ***, p<0.001; **, p=0.001 to p<0.01; *, p=0.01 to p<0.05.

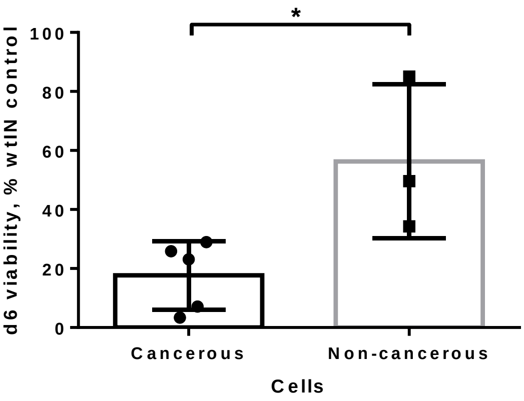

Figure S4: Comparison of the end-viabilities between cancerous and non-cancerous cells. The figure shows the average viability percentages (mean ± SD) of different cells after the 10 ng of p24 LVV IN-I-PpoI dose at day 6 (end of follow-up time, values from Fig. S2). 293T cells are omitted from the analysis due to their altered response. Day 6 viability averages for cancer cells 17.7% ± 5.2 n=5 , and averages of the non-cancerous cells 56.3% ± 15.0 n=3, p<0.001. The difference between groups was analyzed using the unpaired t-test with Welch's correction.
